# *Pseudomonas phaseolicola* preferentially modulates genes encoding leucine-rich repeat and malectin domains in the bean landrace G2333

**DOI:** 10.1007/s00425-022-03943-x

**Published:** 2022-06-29

**Authors:** Paula Rodrigues Oblessuc, David F. Bridges, Maeli Melotto

**Affiliations:** 1 Department of Plant Sciences, University of California, Davis, CA USA; 2 Department of Protection of Specific Crops, InnovPlantProtection Collaborative Laboratory, Elvas, Portalegre, Portugal; 3 Plant Biology Graduate Group, University of California, Davis, CA USA

**Keywords:** Candidate gene expression, *Phaseolus vulgaris*, *Pseudomonas syringae* pv. *phaseolicola*, Plant immunity, Transcriptome

## Abstract

**Main conclusion:**

Candidate resistance genes encoding malectin-like and LRR domains mapped to halo blight resistance loci throughout the common bean genome are co-expressed to fight a range of *Pph* races.

**Abstract:**

Common bean (*Phaseolus vulgaris* L.) is an important crop both as a source of protein and other nutrients for human nutrition and as a nitrogen fixer that benefits sustainable agriculture. This crop is affected by halo blight disease, caused by the bacterium *Pseudomonas syringae* pv. *phaseolicola* (*Pph*), which can lead to 45% yield losses. Common bean resistance to *Pph* is conferred by six loci (*Pse-1* to *Pse-6*) and minor-effect quantitative trait loci (QTLs); however, information is lacking on the molecular mechanisms implicated in this resistance. Here, we describe an in-depth RNA-sequencing (RNA-seq) analysis of the tolerant G2333 bean line in response to the *Pph* strain NPS3121. We identified 275 upregulated and 357 downregulated common bean genes in response to *Pph* infection. These differentially expressed genes were mapped to all 11 chromosomes of *P. vulgaris*. The upregulated genes were primarily components of plant immune responses and negative regulation of photosynthesis, with enrichment for leucine-rich repeat (LRRs) and/or malectin-like carbohydrate-binding domains. Interestingly, LRRs and malectin genes mapped to the same location as previously identified *Pph* resistance loci or QTLs. For instance, the major loci *Pse-6*/HB4.2 involved in broad-resistance to many *Pph* races co-located with induced LRR-encoding genes on Pv04. These findings indicate a coordinated modulation of genes involved in pathogen perception and signal transduction. In addition, the results further support these LRR/malectin loci as resistance genes in response to halo blight. Thus, these genes are potential targets for future genetic manipulation, enabling the introduction of resistance to *Pph* into elite cultivars of common bean.

**Supplementary Information:**

The online version contains supplementary material available at 10.1007/s00425-022-03943-x.

## Introduction

Common bean (*Phaseolus vulgaris* L.) is one of the most important legumes for human nutrition worldwide. The global production of common bean in 2018 was estimated to be 25.4 million tons, with India, Myanmar, Brazil, and United States being the four largest producers (FAOSTAT 2018, http://faostat.fao.org/). Common bean is the main source of protein in many countries in Latin America, Africa, and parts of Asia. It is also rich in complex carbohydrates, vitamins, dietary fiber, and biologically important minerals, such as Ca, Mg, K, Cu, Fe, Mg, and Zn (Blair 2013; Broughton et al. 2003; Foyer et al. 2016). As a legume, common bean also helps to improve soil fertility while reducing the use of synthetic fertilizers, thus promoting sustainable agriculture through symbiotic nitrogen fixation.

Among the many diseases that affect this crop, halo blight, caused by the bacterium *Pseudomonas syringae* pv. *phaseolicola* (*Pph*), can lead to yield losses up to 45% in both temperate and cold climates (Asensio et al. 2006; Singh and Schwartz 2010; Félix-Gastélum et al. 2016). Halo blight is a seed-borne disease and cool, humid environments favor its transmission.

Genetic resistance provides a feasible control of halo blight and is essential to produce disease-free seeds reliably, especially where smallholder farmers rely on seeds saved from a previous harvest (Asensio et al. 2006; Arnold et al. 2011; Miklas et al. 2014). To obtain disease resistant cultivars, it is important to use genetic markers linked to resistance loci identified through linkage mapping analyses. Thus far, six resistance (R) loci have been found (*Pse-1* to *Pse-6*), consisting of five dominant loci and the recessive *pse-*5, which control one or more of the nine *Pph* races. *Pse-1*, *Pse-2*, and *Pse-4* are located on *P. vulgaris* chromosome Pv10; *Pse-3* on Pv02; and *Pse-6* on Pv04 (Miklas et al. 2009, 2011, 2014). None of these race-specific loci provide resistance to the globally prevalent *Pph* race 6, which continues to threaten bean production worldwide (Félix-Gastélum et al. 2016). However, quantitative resistance to *Pph* race 6 is present in a few *P. vulgaris* genotypes, such as CAL 143 and PI 150,414 (Chataika et al. 2011; Tock et al. 2017). Furthermore, minor-effect quantitative trait loci (QTLs) for resistance to *Pph* have been described and may have a crucial role in resistance not only against race 6, but also races 1, 5, 7, and 9 (Trabanco et al. 2014; González et al. 2016, 2017; Tock et al. 2017).

Although genetic mapping is an important tool for crop breeding, genomic and biochemical approaches enable a deeper understanding of the molecular mechanism implicated in plant and pathogen interactions. Applying new generation “omics” technologies is essential to furthering our understanding of the interaction between *P. vulgaris* and *Pph*. Transcription profiles of *Pph* NPS3121 grown in leaf extract and apoplastic fluid of a susceptible bean genotype showed interesting patterns. For instance, genes involved in the first stages of bacterial–plant interactions and pathogen virulence that encode for type III secretion system proteins, cell wall degradation and phaseolotoxin biosynthesis enzymes, and proteins involved in aerobic metabolism were induced, while genes encoding proteins involved in the uptake and metabolism of iron were repressed during *Pph* NPS3121 compatible interaction with common bean (Hernández-Morales et al. 2009). Differential expression of genes associated with respiration, specifically genes in the tricarboxylic acid cycle (TCA) cycle, aerobic respiration, and acetyl-CoA metabolism, were observed when comparing non-piliated with super-piliated type-IV *Pph* mutants, indicating that loss of bacterial mobility might lead to a lower energy draw, thus a differential energy balance among the pathogen and host (Sistrom et al. 2015). Metabolomic analysis of the leaf apoplast wash fluid during compatible (*Pph* RJ3) and incompatible (*Pph* 1302A) interactions with the bean cultivar Tendergreen revealed rapid changes (within the first 10 h post-inoculation) in the chemical composition of the leaf apoplast in both types of interactions. These findings indicate that *Pph* is highly adapted to the leaf apoplast and preferentially uses malate, glucose, and glutamate as a carbon source (O’Leary et al. 2016).

In this study we obtained a holistic overview of the *P. vulgaris* response to *Pph* using Illumina technology to study the transcriptional regulation in the tolerant G2333 landrace during its interaction with the bacterium.

## Materials and methods

### Plant materials and growth conditions

Seeds of the *P. vulgaris* landrace G2333, Jalo EEP 558, CAL 143, and AND 227 were germinated on water-soaked filter paper for 3 days to ensure uniform germination at room temperature. Seedlings were transplanted to individual 42 mm peat moss pellets (Jiffy-7®) and kept at 28 ± 2 °C, 100 ± 10 µmol m^−2^ s^−1^ with a 12-h photoperiod, and 60 ± 10% air relative humidity. Two-week-old plants were used in the experiments detailed below.

### Bacterial pathogenesis assay and sampling

*Pseudomonas syringae* pv. *phaseolicola* (Burkn.) Downs (*Pph*) strain NPS3121 (Peet et al. 1986) was streaked from frozen glycerol culture stock on low-salt Luria Bertani (LSLB) agar plates (10 g/L tryptone, 5 g/L yeast extract, 5 g/L NaCl, pH 7.0, 12 g/L agar), supplemented with 100 μg/mL rifampicin, and incubated at 30 °C for 2–3 days. An individual colony was placed in 100 mL of LSLB liquid medium with 100 μg/mL rifampicin and incubated in an orbital shaker (30 °C and 200 rpm). The bacterial culture was grown until it reached the optical density at a wavelength of 600 nm (OD_600_) between 0.8 and 1.0, as measured by spectrometry. The volume of the bacterial solution reached an inoculum concentration of 1 × 10^6^ CFU/mL and was then centrifuged at room temperature at 1200 g for 20 min. The supernatant was discarded and the bacterial cell pellet was resuspended in sterile distilled water to make the inoculum. The inoculum concentration was assessed by counting colony forming units (CFU) on agar plates. Fully expanded primary leaves were syringe-infiltrated with bacterial inoculum or water as a mock control. After inoculation, plants were grown under the same conditions as described above until the time of sampling. To enumerate the pathogen population in leaves, samples were collected on the day of inoculation (day 0) and at 7 day post-inoculation (dpi) using the protocol described by Jacob et al. (2017). Briefly, leaves were surface sterilized for a serially dilution plate assay. CFU from three independent biological replicates (i.e., three plants) were averaged, log-transformed, and changes in bacterial population (0 versus 7 dpi) were analyzed via paired Student’s *t* tests with *α* = 0.05 as the threshold for significance.

Transcriptional reprogramming is one of the earliest (min to h) plant responses to pathogens (Zipfel and Robatzek 2010). Thus, we collected G2333 leaf samples at 6 and 12 h post-inoculation (hpi) for transcriptome analysis to capture early responses in this system. The experimental design included 16 G2333 plants [2 treatments (mock and bacterium inoculation) × 2 plants per biological replicate × 4 biological replicates], in which four leaves from two different plants represent a biological replicate. Leaves from the same plant were collected at each timepoint (6 and 12 hpi), flash frozen in liquid nitrogen, and stored at − 80 °C until processing. The two leaves collected per timepoint were pooled for total RNA extraction, and RNA from two different plants were pooled for RNA sequencing. The experiment was repeated twice, resulting in four biological replicates.

For gene expression analysis (reverse transcriptase–quantitative polymerase chain reaction, RT-qPCR), leaves from all four bean genotypes were collected at 12 hpi, flash frozen in liquid nitrogen, and stored at − 80 °C until further processing. The experimental design included six plants for each genotype [2 treatments (mock and bacterium inoculation) × 1 plant per biological replicate × 3 biological replicates], in which one leaf from one plant represents a biological replicate.

### Total RNA extraction, TruSeq RNA library preparation, and RNA-sequencing

Total RNA was extracted with the TRIzol reagent (Thermo Fisher Scientific, Rockford, IL, USA) following the manufacturer’s recommendations. RNA was quantified using a NanoDrop spectrophotometer (Thermo Fisher Scientific). RNA quality was evaluated with a BioAnalyzer 2100 (Agilent, Santa Clara, CA, USA). RNA-seq libraries were prepared with 2 µg of total RNA using the TruSeq RNA v2 kit (Illumina Inc., San Diego, CA, USA), according to the manufacturer’s protocol. The concentration of the libraries was measured with the Qubit fluorometer (Invitrogen, Carlsbad, CA, USA) and fragment sizes were determined using the Agilent High Sensitivity DNA kit on a BioAnalyzer 2100 (Agilent, Waldbronn, Germany).

RNA sequences (1 × 100 bases) were obtained with a HiSeq 2000 system (Illumina Inc.) at the DNA Core Facility, University of Missouri, USA. Sequence reads were subjected to a multi-phase quality control regime as follows: raw reads were trimmed with fastx_trimmer using a minimum quality threshold of 13 and minimum length of 32 bases. Subsequently, reads were filtered with a fastq_quality_filter with a quality cutoff of 13 and minimum percentage of 90. Mitochondrial and plastid genome reads and repeat elements were further filtered out using the bowtie-based TopHat suite (Trapnell et al. 2009). Reads that passed quality control were mapped to the *Pph* 1448A genome (GenBank assembly accession: GCA_000012205.1) to remove bacterial sequences, and to the current *P. vulgaris* reference genome assembly v2.1 (https://phytozome.jgi.doe.gov) using the default parameters of the STAR RNA-Seq aligner version 2.5.2b (Dobin et al. 2013). For each biological replicate, an average of 15 million reads were mapped to the *P. vulgaris* genome.

### Differentially expressed gene analysis

The number of reads assigned to each gene was determined and read counts were normalized with the log_2_ counts per million (CPM) normalization method (Law et al. 2016). Similar to a previously described analysis (Oblessuc et al. 2020), the log_2_ fold change (FC) value for each gene was calculated with its normalized expression in *Pph*- versus mock-inoculated samples, and DEG were called based on the Z-ratio method (Cheadle et al. 2003). The Z-ratio approach determines which genes have significantly higher FC than other genes in the data set. A Z-ratio cutoff of 2.25 was used to call approximately 2% of the genes in each data set as being differentially expressed.

DEGs were categorized based on their GO annotations using the Singular Enrichment Analysis tool from AgriGO v2.0 and the reference data set gene model from Phytozome’s *P. vulgaris* reference genome v1.0 (http://systemsbiology.cau.edu.cn/agriGOv2/index.php; Tian et al. 2017). Statistical significance was detected with Fisher’s exact test followed by the Yekutieli-False Discovery Rate multiple test correction (FDR < 0.05). Protein domain enrichment analysis was performed using Phytozome’s PhytoMine with the “Analyze” gene list tool. Venn diagrams were obtained using the Bioinformatics and Evolutionary Genomics web tool (http://bioinformatics.psb.ugent.be/webtools/Venn/). Putative protein orthologs were identified using the NCBI’s BASTp tool (https://blast.ncbi.nlm.nih.gov/) and the *Arabidopsis thaliana* nr database (taxid:3702; *E* value < 1 × 10^–31^). Heat maps were obtained using R-studio software (RStudio Team 2020) and the packages “pheatmap” and “RColorBrewer,” with cluster_cols = TRUE.

### Gene mapping and phylogeny

The physical map was obtained based on the position of *P. vulgaris* genes in Gbrowse v2.1, Phytozome (https://phytozome.jgi.doe.gov) and created with MapChart software (Voorrips 2002). A phylogenetic analysis was performed using MEGAX 10.1 software with clustering via the neighbor-joining method and 1000 bootstraps (Stecher et al. 2020). The complete predicted protein sequences were aligned using the ClustalW algorithm, with gaps larger than five amino acids among all proteins.

### RNA extraction and RT-qPCR

Total RNA was extracted from leaves using the RNAeasy Plant mini kit (Qiagen, Valencia, CA, USA) following the manufacturer’s recommendations with an additional on-column DNase 1 (Qiagen) treatment. Extracted RNA was quantified using a NanoDrop spectrophotometer (Thermo Scientific) and then reserve-transcribed using the Takara RNA PCR kit (Clontech, Mountain View, CA, USA), 150 ng/μL of total RNA, and 0.125 μM of oligo-dT primer, according to the manufacturer’s protocol. The reverse-transcription (RT) reaction was performed at 50 °C for 30 min, followed by 95 °C for 5 min. Quantitative PCR (qPCR) reactions were carried out using the iTaq Fast SYBR green supermix (BioRad, Hercules, CA, USA) reagents per manufacturer’s instructions, 6.5 ng/mL cDNA, and 500 nM gene-specific primers in a final volume of 10 μL. qPCR cycles consisted of one cycle of 95 °C for 5 min, 40 cycles of 95 °C for 10 s, 60 °C for 30 s, followed by the dissociation-curve default parameters using the BioRad CFX Real-Time PCR System. Gene expression levels in *Pph*-infiltrated plants were compared to that of mock-infiltrated plants using the 2^−ΔΔCq^ method (Livak and Schmittgen 2001) and log_2_ transformed. The *P. vulgaris INSULIN DEGRADING ENZYME* (*PvIDE*; Phvul.001G133200) was used as a reference gene (Borges et al. 2012) and the target genes included in the analysis were Phvul.004G008740, Phvul.004G015800, Phvul.005G162600, Phvul.008G030800, and Phvul.008G164300. Differences in expression of the selected genes in the infected plants compared to the mock control were analyzed via paired Student’s *t* tests with *α* = 0.05.

### Data availability

The Illumina RNA-seq data related to this study is available at the NCBI Gene Expression Omnibus (GEO) under accession number GSE173535 (https://www.ncbi.nlm.nih.gov/geo/query/acc.cgi?acc=GSE173535).

## Results

**Fig. 1:**
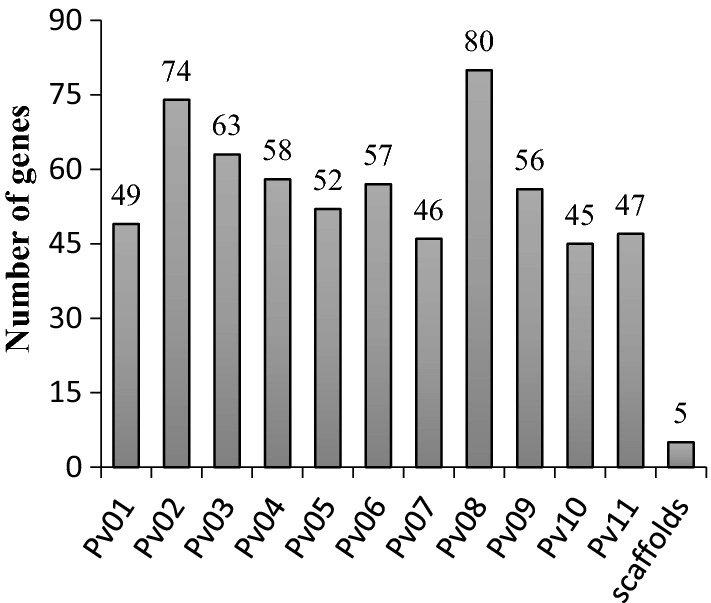
Mapping of common bean genes modulated by *Pseudomonas syringae* pv. *phaseolicola*. Distribution of differentially expressed genes (DEGs) throughout the *P. vulgaris* genome. DEGs (total of 632) mapped to all 11 *P. vulgaris* chromosomes and scaffolds (genome version v2.1; https://phytozome.jgi.doe.gov/). The number on top of the bars indicates the total DEGs mapped to each genome region

Our genome-wide transcriptome profiling allowed for the detection of 17,870 unique transcripts in the leaf samples (Table S1), indicating a normal distribution of expression based on the Z-ratio values (Fig. S1a), which had a strong correlation with the log_2_ FC values (Fig. S1b). Based on the Z-ratio analysis, 275 and 357 were significantly upregulated and downregulated, respectively, in samples inoculated with *Pph* (Table S2). Approximately 25% of these DEGs mapped to the common bean chromosomes Pv02 and Pv08, with the remaining 75% mapped to the remaining chromosomes and scaffolds (Fig. 1).

The GO enrichment analysis (Table S3) showed an over-representation of biological process and molecular function terms related to plant immune responses. In particular, programmed cell death and apoptosis processes were enriched among upregulated genes, which are well known to be activated during the hypersensitive response against pathogens (reviewed by Balint-Kurti 2019). Enrichment for signal transduction through post-translational protein modifications, such as phosphorylation, kinase and transferase activities, transferring phosphorus-containing groups, adenyl ribonucleotide binding, adenyl nucleotide binding, and nucleoside binding were also observed (Fig. S2). Furthermore, the upregulated genes were mainly involved in photosynthesis as part of the thylakoid cellular component. However, the genes in these GO categories are annotated as negative regulators of the light reaction and photosynthetic electron transport chain at the photosynthetic membrane and photosystem, indicating the induction of regulation to reduce photosynthetic rates (Fig. S2). Genes downregulated by *Pph* inoculation were primarily in the GO categories involved in oxidation, reduction, and catalytic activity metabolic processes at the ribosome (Fig. S3), indicating that the repression of oxidative reactions is a result of the negative regulation of photosynthesis.

**Fig. 2:**
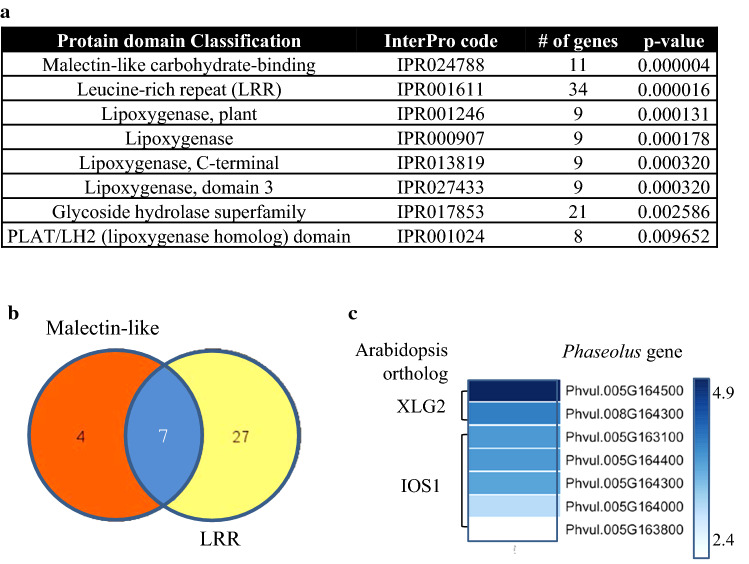
Protein domain enrichment analysis. **a** List of enriched protein domains (Holm–Bonferroni; > 5 genes and *p* < 0.01) identified among DEGs according to InterPro codes (http://www.ebi.ac.uk/interpro). **b** Venn diagram showing DEGs that encode leucine-rich repeat (LRR) and/or malectin domains. **c** Heatmap of expression levels (Z-ratio) of the seven DEG containing both malectin-like and LRR domains and their putative Arabidopsis orthologs (*E* value = 0.0, Identity > 45%)

Next, we identified the protein domains encoded by the DEGs. This analysis revealed an overrepresentation of malectin-like carbohydrate-binding, leucine-rich repeat (LRR), lipoxygenase, and glycoside hydrolase superfamily domains (Fig. 2a). Among the proteins with malectin-like carbohydrate-binding and LRR domains (Table S4), seven are predicted to contain both domains, four have only the malectin-like domain, and 27 have only the LRR domain (Fig. 2b). Sequence analysis showed that two genes induced by *Pph* (Z-ratio > 3.8) encode for proteins with both malectin-like and LRR domains (Phvul.005G164500 and Phvul.008G164300) and are putative orthologs of *Arabidopsis thaliana* XLG2 (EXTRA-LARGE GUANINE NUCLEOTIDE-BINDING PROTEIN 2). The other five proteins containing both malectin-like and LRR domains (Phvul.005G163100, Phvul.005G163800, Phvul.005G164000, Phvul.005G164300, and Phvul.005G164400) are putative orthologs of the *A. thaliana* IOS1 (IMPAIRED OOMYCETE SUSCEPTIBILITY 1) protein. The *P. vulgaris* genes that code these seven proteins showed a narrow range of expression (Z-ratios from 2.4 to 4.9), indicating possible co-regulation (Fig. 2c).

**Fig. 3:**
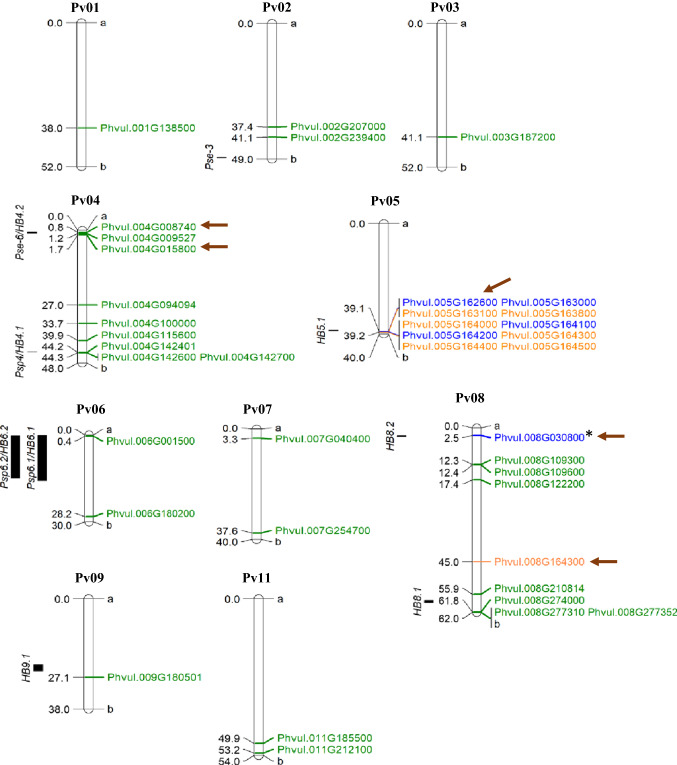
Chromosome location of the 38 common bean genes encoding malectin-like and/or LRR domains regulated by *Pph*. The numbers on the left are the physical positions of each gene in the common bean chromosomes (in Mb). Letters “a” and “b” indicate the beginning and end of the chromosome, respectively. Genes tagged with green, blue, or orange code for LRR domain, malectin-like domain, or proteins with both LRR and malectin-like domains, respectively. The black rectangles on the left of the chromosomes indicate the physical position of the previously mapped *Pph* resistance QTLs (Miklas et al. 2014; Trabanco et al. 2014; Tock et al. 2017). Brown arrows point to genes that had their expression assessed via RT-qPCR. The gene Phvul.008G030800 (*) was previously described (Oblessuc et al. 2015). The map was created using MapChart software (Voorrips 2002)

**Fig. 4:**
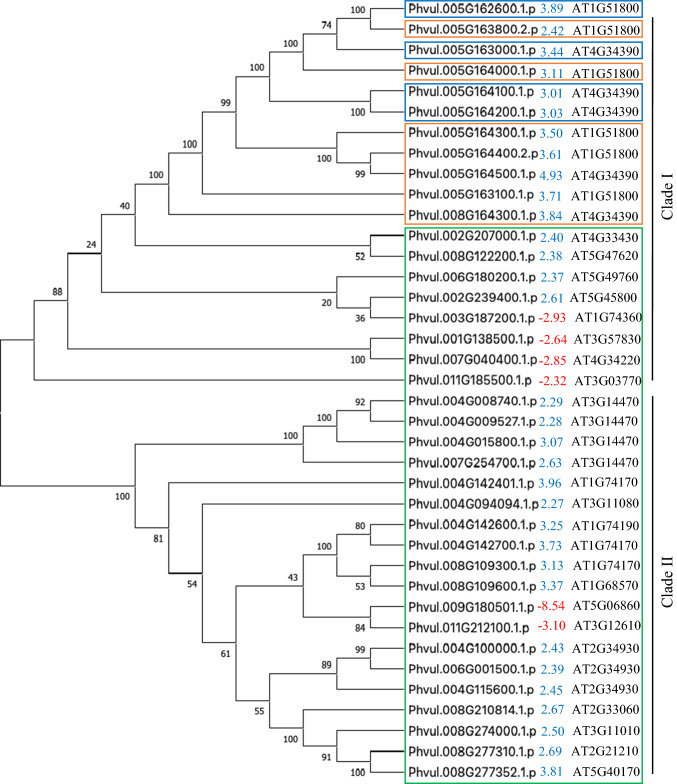
Phylogenetic analysis of DEGs encoding malectin and/or LRR domains. Complete predicted proteins were aligned using the ClustalW algorithm in MEGAX. Phylogenetic clustering was accomplished using the neighbor-joining method with 1000 bootstraps. The number in front of the gene ID is the Z-ratio differential expression, wherein red and blue fonts indicate downregulation and upregulation, respectively, upon bacterium infection. The putative *Arabidopsis thaliana* ortholog (*E* value < 10^–31^) for each *P. vulgaris* protein is listed on the right. Genes tagged with green, blue, and orange squares code for LRR domain, malectin-like domain, and proteins with both LRR and malectin-like domains, respectively

Mapping of the 38 genes encoding for malectin and/or LRR proteins revealed that the LRR proteins are located in all chromosomes except Pv05 and Pv10 (Fig. 3). Interestingly, genes coding for proteins with a malectin-like domain alone or proteins with a malectin and an LRR domains formed a cluster in Pv05, which co-located with the previously mapped QTL HB5.1 (Tock et al. 2017) (Fig. 3). Only one malectin–LRR encoding gene was located on another chromosome (Pv08; Phvul.008G164300) (Fig. 3), which is phylogenetically distant from the other LRR genes regulated by *Pph* in this chromosome (Fig. 4). Notably, some LRR genes induced by *Pph* were mapped close to QTLs *Pse-6/*HB4.2 and *Psqp4/*HB4.1 in Pv04, QTLs Psp6.1*/*HB6.1 and Psp6.2*/*HB6.2 in Pv06, and QTLs HB8.1 and HB8.2 in Pv08, while an LRR repressed by *Pph* mapped close to QTL HB9.1 in Pv09 (Fig. 3). Altogether, these results suggest that the common bean response at the early stages of *Pph* infection leads to induction of LRR and malectin domain-encoding genes that are associated with disease resistance described in previous QTL mapping studies (Miklas et al. 2014; Trabanco et al. 2014; Tock et al. 2017).

Phylogenetic clustering analysis of all 38 proteins with LRR and/or malectin domains encoded by *Pph-*regulated genes revealed two major clades: Clade I containing malectin-like and/or LRR domain proteins, and Clade II harboring only LRR proteins (Fig. 4). As expected, proteins with a malectin-like domain clustered in a sub-clade within Clade I that was distinct from proteins containing an LRR domain alone. Interestingly, all of these malectin domain proteins, with exception of Phvul.008G164300.1.p, are located at the end of chromosome Pv05 (Fig. 3). Among the LRR proteins, 19 have a kinase domain, out of which six LRR-kinases were downregulated by *Pph* in G2333. In Clade I, the coded protein Phvul.003G187200.1.p is an ortholog of *A. thaliana* NILR1 (NEMATODE-INDUCED LRR–RLK 1, AT1G74360), Phvul.001G138500.1.p is an ortholog of the Arabidopsis protein AT3G57830, Phvul.007G040400.1.p is an ortholog of Arabidopsis RECEPTOR DEAD KINASE1 (AT4G34220), known to be involved in ABA-mediated seedling development and drought tolerance, and Phvul.011G185500.1.p is an ortholog of AT3G03770. In Clade II, the coded protein Phvul.009G180501 is an ortholog of PGIP1 (POLYGALACTURONASE INHIBITING PROTEIN 1, AT5G06860) and Phvul.011G212100 is an ortholog of DNA-DAMAGE REPAIR Arabidopsis protein (AT3G12610). All other LRR and/or malectin genes were upregulated after *Pph* infection, including Phvul.002G207000.1.p, a putative ortholog of BAK1 (BRASSINOSTEROID INSENSITIVE 1-ASSOCIATED RECEPTOR KINASE 1, AT4G33430) that is an important co-regulator of plant immunity and growth (Fig. 4 and Table S4).

**Fig. 5:**
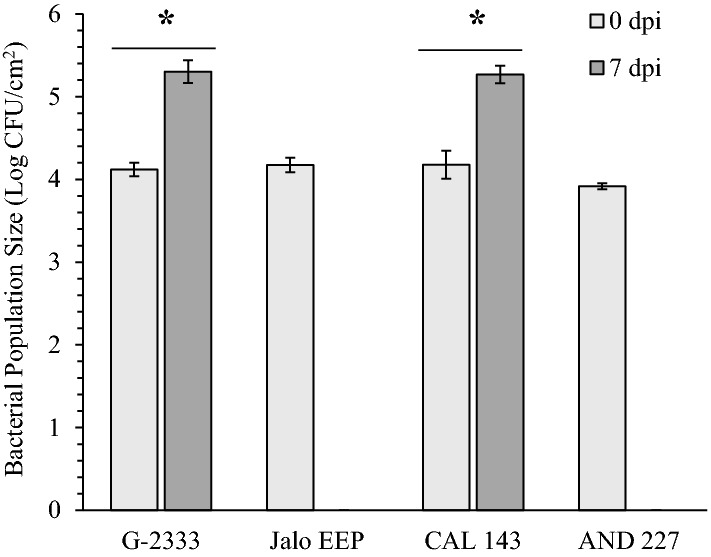
*Pseudomonas syringae* pv. *phaseolicola* growth in four common bean genotypes over 7 days. Data are presented as means ± standard error (*n* = 3). Significant differences in bacterial populations between 0 and 7 day post-inoculation (dpi) were measured via paired Student’s *t* test (*α* = 0.05) and are indicated with an asterisk (*). Bacterial cells were not recovered from the resistant genotypes Jalo EEP and AND 227 at 7 dpi; thus, statistical tests could not be performed for these genotypes

**Fig. 6:**
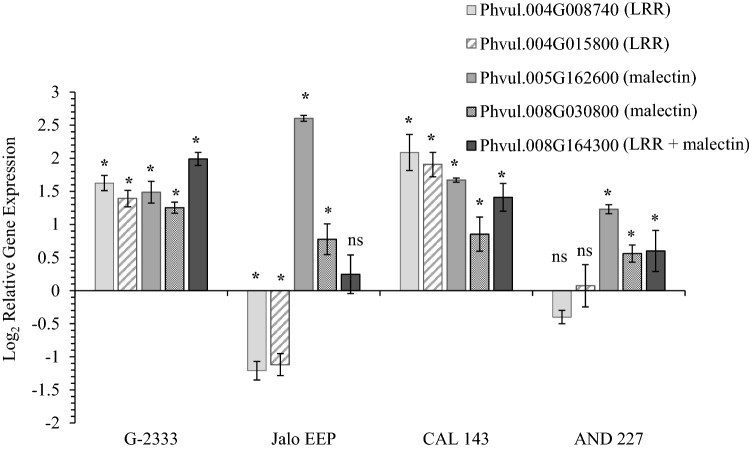
Relative expression of select genes in common bean genotypes inoculated with *P. syringae* pv. *phaseolicola*. Gene expression at 12 hpi was determined by RT-qPCR. Gene expression data were analyzed using the 2^−ΔΔCq^ method, log_2_ transformed, and are presented as means ± standard error (*n* = 3). Significant differences in the expression of each gene in bacterium-inoculated plants compared to mock control were determined via paired Student’s *t* test (*α* = 0.05) and are signified by an asterisk (*). *ns* non-significant

To start validating the function of DEGs during common bean reaction to *Pph*, we performed RT-qPCR analysis using gene specific primers (Table S5) for selected DEGs that mapped closely to previously identified QTLs (Fig. 3). For this analysis, we included four bean genotypes that showed either strong resistance (Jalo EEP and AND 227) or tolerance (G2333 and CAL 143) to the *Pph* (Fig. 5). Bacterial cells were not recovered from Jalo EEP and AND 227 at 7 dpi, whereas *Pph* population grew approximately 1.5 log in G2333 and CAL 143 (Fig. 5) without causing visible symptoms (data not shown). Confirming the RNA-seq analysis, all five genes tested were significantly upregulated in the *Pph*-tolerant genotypes G2333 and CAL 143 at 12 hpi. However, different expression patterns were observed in *Pph*-resistant genotypes according to the genes analyzed (Fig. 6). The genes Phvul.004G008740 and Phvul.004G015800, which map in the vicinity of *Pse-6/*HB4.2 and encode for an LRR domain, were significantly downregulated in Jalo EEP, but not regulated in AND 227, suggesting that the involvement of these genes in resistance to *Pph* strain NPS3121 may be dependent on the plant genetic background. The gene Phvul.005G162600 that maps in the vicinity of HB5.1 and the gene Phvul.008G030800 that maps close to HB8.2, both encoding for a malectin-like domain, were significantly upregulated in all genotypes, suggesting that they might be associated with general plant responses to this bacterium. The gene Phvul.008G164300 that encodes for both LRR and malectin-like domains was not significantly regulated in Jalo EEP, whereas it was significantly upregulated in AND 227, suggesting that this gene may be relevant to genotype-specific resistance to *Pph*.

## Discussion

The common bean landrace G2333 is tolerant to *Pph* strain NPS3121, as its leaves support a high bacterial population, typical of susceptible interactions, but without visible symptoms that are typical of resistant cultivars (Fig. 5; Oblessuc et al. 2015). At the early stages of *Pph* NPS3121 infection (6 and 12 hpi), G2333 showed a complex response with a great number of genes regulated throughout its genome (Fig. 1). This intense transcriptional regulation is an expected plant response that has been reported for many host–pathogen interactions (reviewed by Wilkinson et al. 2019). The repression of photosynthesis by chloroplast-targeted effectors and phytotoxins is also a common plant response to pathogens (Lu and Yao 2018). Photosynthesis is essential for plant defense by generating ATP, NADPH, and carbohydrates used by the plant to synthesize primary metabolites, defense-related hormones, and other antimicrobial compounds. In addition, chloroplasts produce reactive oxygen species (ROS) and nitric oxide, which are essential molecules for plant defense (reviewed by Lu and Yao 2018). ROS production is induced during hypersensitive response (HR), localized programmed cell death (PCD), which is a hallmark of common bean resistance to *Pph* that acts via minimizing the pathogen spread throughout the leaf (Tock et al. 2017). We observed that *Pph* inoculation represses photosynthesis and oxidation reduction processes (Fig. S2 and S3), while no water-soaked symptoms of HR were observed in G2333 (Oblessuc et al. 2015). Nonetheless, *Pph* also induced genes involved in apoptosis (Fig. S2), a type of PCD (Locato and Gara 2018). Together, these findings suggest that *Pph* might repress photosynthesis and ROS production in G2333 to prevent plant HR and promote bacterial proliferation, while plant defense responses are activated, resulting in apoptosis to control extensive bacterial spread and disease symptoms development.

In addition to physiological alterations, the defense responses in G2333 may be related to the function of LRR and malectin proteins, as indicated by the enrichment of these domains in predicted proteins coded by genes primarily induced by *Pph* (Fig. 2). LRR proteins are traditionally involved in plant immunity by directly or indirectly recognizing effectors secreted by pathogens, activating downstream signaling pathways that lead to the expression of defense genes (Miklas et al. 2014; Meziadi et al. 2016). The common bean genome has three major clusters of LRR proteins located at the ends of chromosomes Pv04, Pv11 (mainly CC–NB–LRR proteins), and Pv10 (enriched for TIR–NB–LRR proteins) (Meziadi et al. 2016). Interestingly, *Pph* inoculation regulated the transcription of LRR genes in chromosomes Pv04 and Pv011, but not those located in Pv10 (Fig. 3), indicating that while the CC–NB–LRR sub-group of R genes has a role in the G2333 response to *Pph*, the TIR–NB–LRR genes might not.

QTLs involved in common bean resistance to *Pph* were previously mapped to the genomic regions containing LRR genes identified here (González et al. 2016, 2017; Tock et al. 2017; Fig. 3). *Pse-6* confers common bean resistance to *Pph* races 1, 5, 7, and 9 and was mapped to the proximal end of Pv04, linked to the SCAR marker SB10.550 (Miklas et al. 2014). LRR proteins in Pv04 are known to confer resistance not only to *Pph* but also to Bean Golden Yellow Mosaic Virus (BGYMV), *Colletotrichum lindemuthianum* that causes anthracnose (locus *Co*-*3*), *Macrophomina phaseolina* that causes ashy stem blight, and *Uromyces appediculatus* that causes bean rust (Geffroy et al. 2000; Miklas et al. 2006; Ferreira et al. 2013). Moreover, a major QTL, named HB4.2, was mapped between 0.6 and 2.1 Mb of Pv04, conferring broad-spectrum resistance to eight *Pph* races (Tock et al. 2017). Based on both RNA-seq and RT-qPCR analyses, we observed the upregulation of Phvul.004G008740 and Phvul.004G015800 in G2333 leaves after *Pph* inoculation (Table S2; Fig. 6), which are mapped between 0.8 and 1.7 Mb of Pv04, co-locating with *Pse-6* and HB4.2 and Phvul.004G009527 (Fig. 3). These common bean genes formed a sub-cluster of closely related LRR proteins within Clade II, which grouped other six genes located in chromosome Pv04 (Fig. 4). In fact, this genome region has nine predicted genes coding for LRR proteins that were upregulated by *Pph* (Table S2). These indicate that the *Pse-6*/HB4.2 genome region is a hot spot for gene duplication, which may maintain the same set of functions while increasing diversity, promoting an adaptative advantage in the face of pathogen evolution.

The genes Phvul.004G008740, Phvul.004G009527, and Phvul.004G015800 code for LRR-domain proteins (Fig. 2) similar to AT3G14470, an NB–ARC domain-containing disease resistance protein of Arabidopsis (Table S4). The NB–ARC domain proteins are R proteins that, among other functions, were shown to promote cell death in plants (Van Ooijen et al. 2008). According to the Phytozome *Phaseolus vulgaris* v2.1 gene annotation, all three proteins are classified in the EuKaryotic Orthologous Group Apoptotic ATPase (KOG4658). Moreover, Phvul.004G008740 and Phvul.004G015800 were upregulated in G2333 and CAL 143 but not in Jalo EEP and AND 277 (Fig. 6). CAL 143 is an important resistance source in the field against different pathogens, including different *Pph* races, in which HB4.2 was mapped (Tock et al. 2017). Altogether, these indicate that *Pse-6*/HB4.2 is a resistance locus containing LRR paralogs co-regulated under pathogen infection to promote race-specific resistance, possibly through cell apoptosis. These results also suggest that, although Jalo EEP and AND 277 are highly resistant to *Pph* (Fig. 5), a different immune mechanism is activated in these genotypes against *Pph* NPS3121.

The QTL HB5.1 for *Pph* resistance is located on Pv05 in the same region as ten G2333 malectin-domain genes regulated by *Pph* (Fig. 3), including Phvul.005G162600 that was also induced in all other common bean genotypes used here (Fig. 6). Malectin domain proteins were recently linked to plant–pathogen interactions and participate in plasma membrane processes that activate downstream signaling to promote the crosstalk between plant growth and defense (Stegmann et al. 2017; Zhang et al. 2020). Moreover, other two malectin domain genes were mapped close to previously found QTLs. Phvul.008G164300 was mapped in the vicinity of the QTL HB8.1, and Phvul.008G030800, named *FERONIA (FER)-like* (Oblessuc et al. 2015), located close to HB8.2 (Tock et al. 2017; Fig. 3). FER is a member of the *Catharanthus roseus* receptor-like kinase1 (RLK1)-like family, known by its carbohydrate-binding activity, which controls plant growth and plant defense in Arabidopsis (Stegmann et al. 2017; Franck et al. 2018). We previously showed that COK-4, a common bean kinase, can complement the function of mutated FER in the defense and growth of Arabidopsis *fer-5* (Azevedo et al. 2018), supporting the notion that common bean has a similar signaling pathway as the one activated by FER in Arabidopsis. HB5.1 also influences seed yield (Tock et al. 2017), encouraging the hypothesis that malectin-domain proteins are involved in the growth-defense tradeoff in various common bean genotypes. Notably, Phvul.008G030800 was previously shown to be induced in the early stages of G2333 infection (Oblessuc et al. 2015) as well as in Jalo EEP, CAL 143, and AND 277, while Phvul.008G164300 was not regulated in Jalo EEP only (Fig. 6). Therefore, one could hypothesize that these three malectin domain genes play a role in broad-resistance against *Pph*, but they should be used with caution in breeding programs as they may also affect growth-related traits, such as yield. Further functional studies would be essential to evaluate which malectin domain genes could uncouple this growth-defense tradeoff, if any.

Phvul.005G162600 and Phvul.008G164300 are among the most induced malectin–LRR genes (Table S2), which together with the other five and four genes at Pv05 are homologs of Arabidopsis IOS1 and XLG2, respectively (Fig. 2; Table S4). XLG2 is known to be a modulator of transmembrane signaling systems that function as a positive regulator of the salicylic acid pathway, in addition to root morphogenesis and flowering (Ding et al. 2008; Zhu et al. 2009; Heo et al. 2012). IOS1 is required for BAK1-dependent and BAK1-independent pattern-triggered immunity (PTI), as well as for full plant susceptibility to oomycetes and fungal pathogens (Hok et al. 2011; 2014; Yeh et al. 2016). Interestingly, the gene Phvul.002G207000, which is functionally annotated as BAK1, was also induced by *Pph* (Table S4). Phvul.002G207000 was mapped to Pv02, near *Pse-3* (Fig. 3). *Pse-3* co-segregates with the *I* gene that confers immune response or temperature-dependent HR to Bean Common Mosaic Virus and a temperature-independent HR to Bean Common Mosaic Necrosis Virus (Miklas et al. 2011). This suggests that the putative IOS1 common bean genes could be involved in bean basal immunity against *Pph* (and potentially other pathogens) through BAK1-mediated signaling and activation of salicylic acid responses through XLG2.

Three minor-effect QTLs, Psp4, Psp6.1, and Psp6.2 (renamed by Tock et al. (2017) as HB4.1, HB6.1, and HB6.2, respectively), mapped to Pv04 at 44 Mb, Pv06 at 0.2–15.8 Mb, and Pv06 0.3–14.9 Mb, respectively (Trabanco et al. 2014; Tock et al. 2017). Three LRR genes (Phvul.004G142401, Phvul.004G142600, and Phvul.004G142700) were upregulated by *Pph* and located close to Psp4/HB4.1, while Psp6.1/HB6.1 and Pps6.2/HB6.2 were mapped close to the also induced gene Phvul.006G001500 (Fig. 3). Therefore, these genes could play a role in the Psp4/HB4.1-, Psp6.1/HB6.1-, and Pps6.2/HB6.2-mediated resistance. Trabanco et al. (2014) suggested five candidate genes located in Pv04 for resistance to *Pph* based on their homology to Arabidopsis and soybean (*Glycine max*) proteins with known functional roles in plant immunity, namely, FLS2 (FLAGELLIN SENSING 2) and Pto. The Arabidopsis *FLS2* gene codes for the flagellin receptor involved in PTI responses to bacterial pathogens (Gómez-Gómez and Boller 2000). The soybean *Pto* gene codes for a serine/threonine kinase that confers resistance to *P. syringae* pv. *tomato* strains that express the avirulence gene *AvrPto* (Chandra et al. 1996). We have previously reported that at early stages of *Pph* infection (6 and 12 hpi) two *FLS2-like* genes (Phvul.002G196200 and Phvul.005G149200) were repressed in G2333 (Oblessuc et al. 2015). Based on the current *P. vulgaris* v2.1 genome annotation, only Phvul.002G196200 is annotated as *FLS2*-*like*. In addition, the genes suggested by Trabanco et al. (2014) as possible homologs of FLS2 showed no such hits in our recent NCBI–BLAST analysis. It is possible that new functional and sequence information led to this variation in results.

Previously, 11 candidate genes within QTLs Psp6.1/HB6.1 and Psp6.2/HB6.2 were suggested to be involved in resistance to *Pph* based on sequence homology (Trabanco et al. 2014). All of them show homology with Arabidopsis *RPM1* that confers resistance to *P. syringae* expressing the avirulence genes *AvrRpm1* and/or *AvrB* (Mackey et al. 2002), and soybean *RPG1-B* that confers resistance to *P. syringae* pv. *glycinea* expressing *AvrB* (Trabanco et al. 2014). In the present study, none of these 11 common bean genes or the five genes on Pv04 (Trabanco et al. 2014) were regulated by *Pph*, suggesting that co-localization of mapped QTLs with genes putatively involved in plant defense based on sequence homology is not enough to infer candidate resistance genes. Alternatively, this difference may be related to the fact that Trabanco et al. (2014) used a different common bean genotype as resistance source (the moderate resistant Cornell 49,242), and as reported here, different genetic backgrounds lead to different resistance phenotypes and gene expression patterns (Figs. 5 and  6). Moreover, a plant’s response to a pathogen involves different patterns of gene expression; therefore, the RNA-seq performed here is a snapshot of the immune response. It is possible that the candidate genes suggested by Trabanco et al. (2014) could be modulated by *Pph* at different timepoints, still participating in the resistance of common bean to *Pph*. These possibilities reinforce the need for further studies, including transcriptome and functional analyses in various genetic backgrounds.

**Fig. 7:**
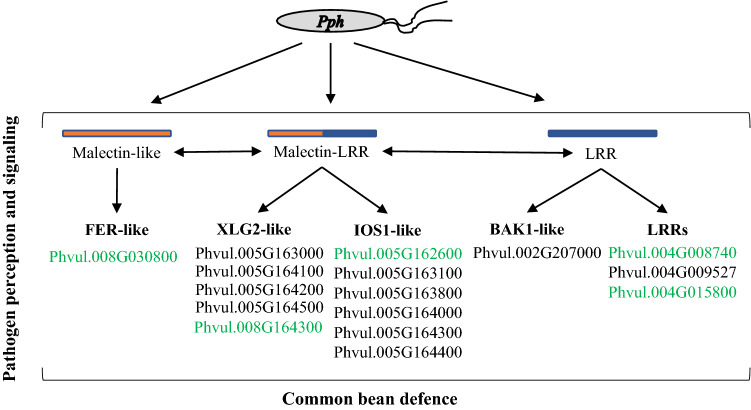
Common bean candidate genes and their encoded protein domains that promote defense against *Pph*. Common bean malectin-domain protein FER-like (Oblessuc et al. 2015), together with the malectin–LRR putative homologs of XLG2 and IOS1 (mapped at the same location as HB5.1; Tock et al. 2017), would work together with BAK1-like and other LRR domain proteins co-localized with the major QTL *Pse-6*/HB4.2 (Tock et al. 2017) to promote plant defenses against *Pph*. Genes in green letters had their expression verified by RT-qPCR

Altogether, the data indicate that LRR proteins, mainly those co-localizing with the major resistance QTL *Pse-6/*HB4.2 in Pv04, would function in race-specific resistance and together with malectin-domain proteins, especially those co-localized with the QTL HB5.1 in Pv05, would promote basal plant immunity against *Pph* and potentially other pathogens. In addition, the QTL HB5.1 was previously shown to influence seed yield and interact with the major *Pph* resistance QTL *Pse-6*/HB4.2 (Tock et al. 2017). Therefore, LRR and malectin genes may act quantitatively in the resistance of common bean to *Pph*, as well as in the balance between growth and defense. Among the malectin proteins close to HB5.1, common bean PvIOS1-like and PvXLG2-like (mapped at Pv05 and Pv08) could function together with PvBAK1-like to regulate plant defenses (Fig. 7). These genes and the pathways associated with their function can be potential targets for enhancing plant defenses to control halo blight disease of common bean.

## Supplementary Information

Below is the link to the electronic supplementary material.

Supplementary file1 (PPTX 725 KB)

Supplementary file2 (PPTX 351 KB)

Supplementary file3 (PPTX 284 KB)

Supplementary file4 (XLSX 1777 KB)

Supplementary file5 (XLSX 51 KB)

Supplementary file6 (XLSX 17 KB)

Supplementary file7 (XLSX 16 KB)

Supplementary file8 (DOCX 14 KB)
