# Supplementary material for: Pseudomonas phaseolicola preferentially modulates genes encoding leucine-rich repeat and malectin domains in the bean landrace G2333
Source: Planta. 2022 Jun 29;256(2):25. doi: 10.1007/s00425-022-03943-x (PMC9242968; doi:10.1007/s00425-022-03943-x)
Supplement: Supplementary file 1 — Supplementary file1 (PPTX 725 KB) [file 425_2022_3943_MOESM1_ESM.pptx]

## Slide 1
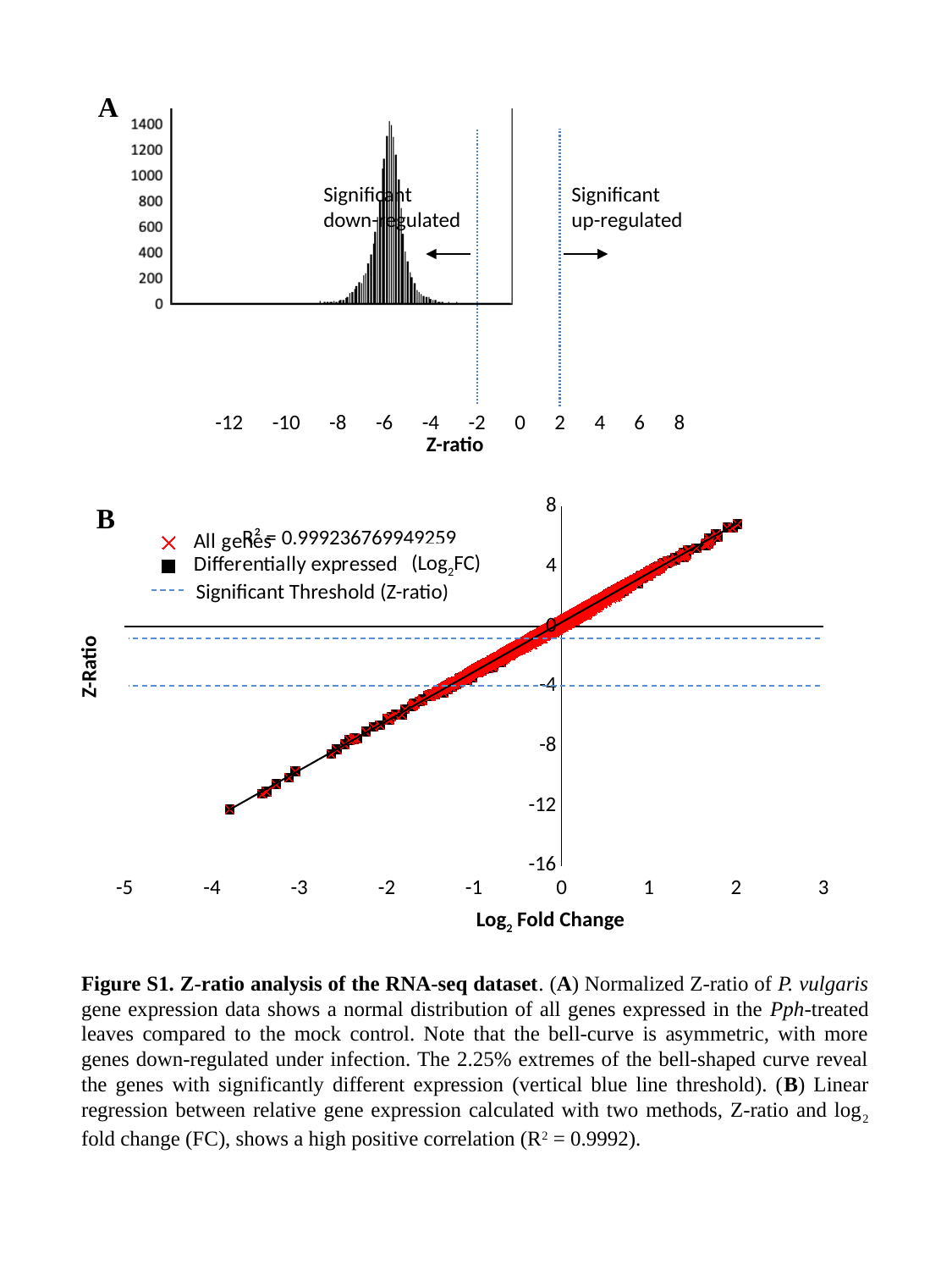

A
-12 -10 -8 -6 -4 -2 0 2 4 6 8
Z-ratio
Significant
up-regulated
Significant
down-regulated
### Chart
| Category | All genes | Differentially expressed (Log2) |
|---|---|---|Significant Threshold (Z-ratio)
(Log2FC)
B
 Z-Ratio
 Log2 Fold Change
Figure S1. Z-ratio analysis of the RNA-seq dataset. (A) Normalized Z-ratio of P. vulgaris gene expression data shows a normal distribution of all genes expressed in the Pph-treated leaves compared to the mock control. Note that the bell-curve is asymmetric, with more genes down-regulated under infection. The 2.25% extremes of the bell-shaped curve reveal the genes with significantly different expression (vertical blue line threshold). (B) Linear regression between relative gene expression calculated with two methods, Z-ratio and log2 fold change (FC), shows a high positive correlation (R2 = 0.9992).
