# Supplementary figures and images for: Pseudomonas phaseolicola preferentially modulates genes encoding leucine-rich repeat and malectin domains in the bean landrace G2333
Source: Planta. 2022 Jun 29;256(2):25. doi: 10.1007/s00425-022-03943-x (PMC9242968; doi:10.1007/s00425-022-03943-x)

## Slide 1
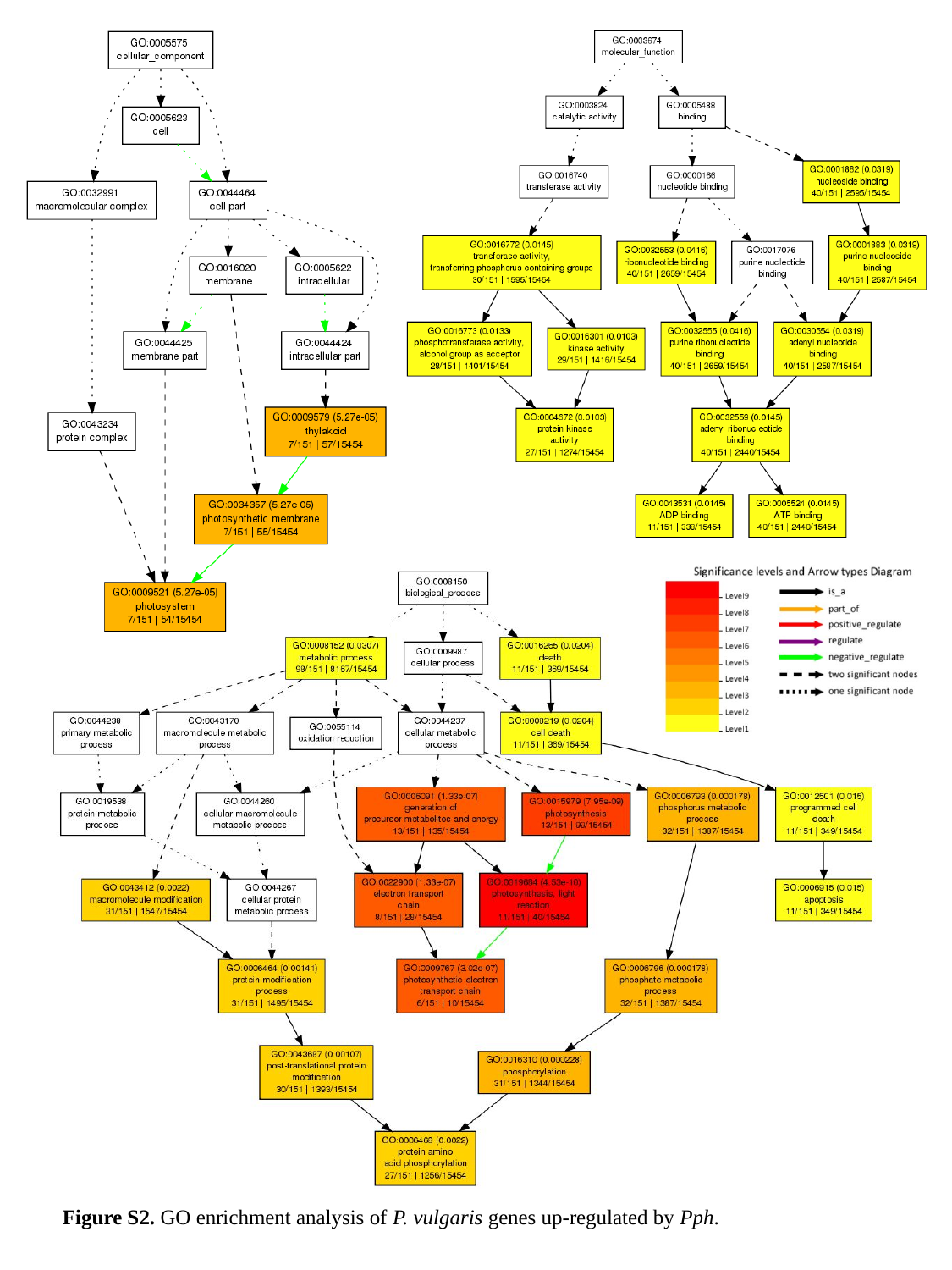

Figure S2. GO enrichment analysis of P. vulgaris genes up-regulated by Pph.

Supplement: Supplementary file 2 — Supplementary file2 (PPTX 351 KB) [file 425_2022_3943_MOESM2_ESM.pptx]

## Slide 1
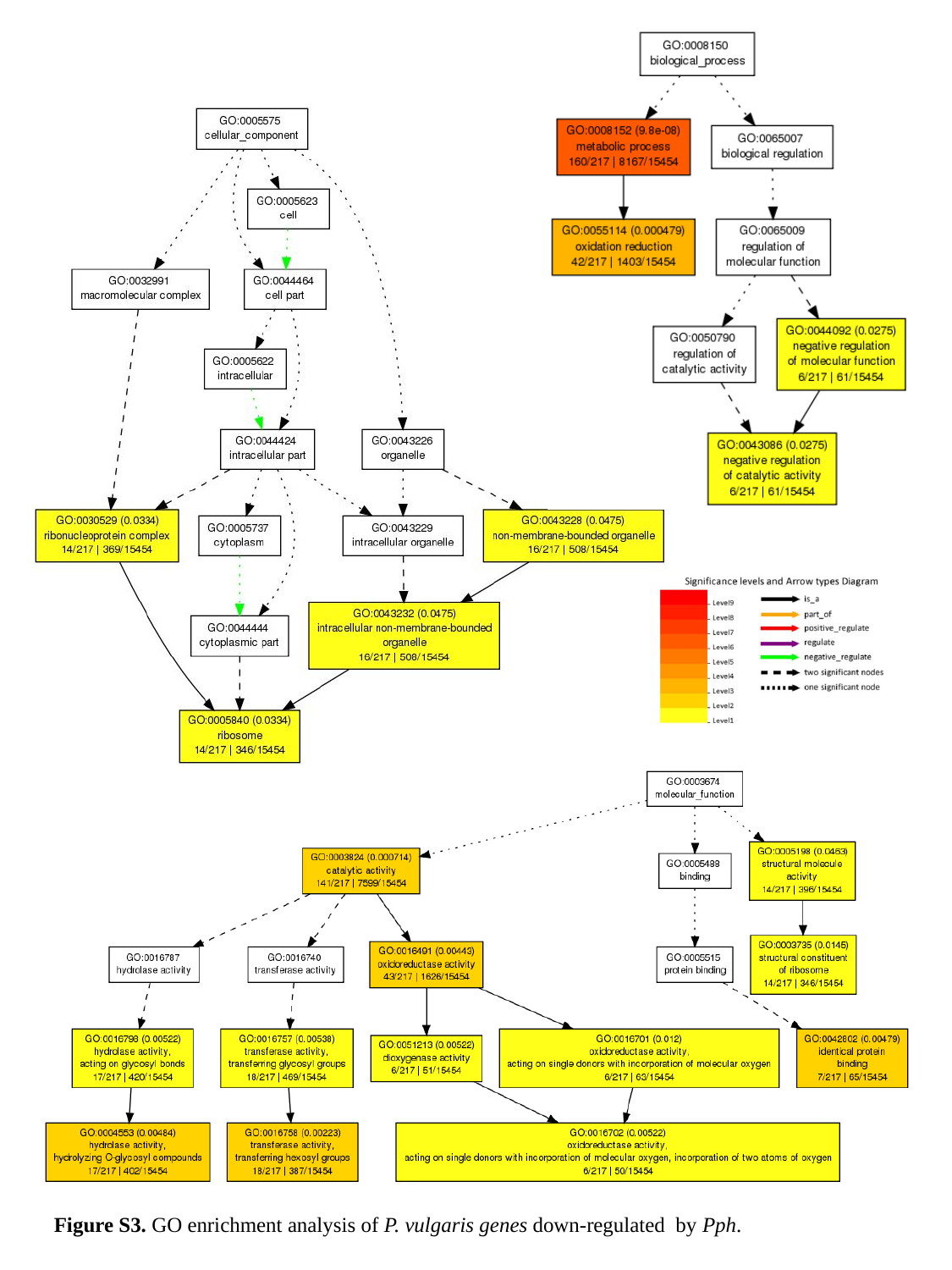

Figure S3. GO enrichment analysis of P. vulgaris genes down-regulated by Pph.

Supplement: Supplementary file 3 — Supplementary file3 (PPTX 284 KB) [file 425_2022_3943_MOESM3_ESM.pptx]
