# Supplementary material for: Pseudomonas phaseolicola preferentially modulates genes encoding leucine-rich repeat and malectin domains in the bean landrace G2333
Source: Planta. 2022 Jun 29;256(2):25. doi: 10.1007/s00425-022-03943-x (PMC9242968; doi:10.1007/s00425-022-03943-x)
Supplement: Supplementary file 8 — Supplementary file8 (DOCX 14 KB) [file 425_2022_3943_MOESM8_ESM.docx]

**Table S5**. Primers used for RT-qPCR reactions. Phvul.001G133200 was used as an internal reference for the gene expression analysis as described by Borges et al. (2011). F= forward primer, R = reverse primer.

| Target | Sequence (5’-3’) |
| --- | --- |
| Phvul.001G133200 | F: GCAACCAACCTTTCATCAGC  R: AGAAATGCCTCAACCCTTTG |
| Phvul.008G030800 | F: GGCTCCTAACTACACTGCACCAGAC  R: GCACAAGCTTTTAGGGTTAGGATGC |
| Phvul.004G008740 | F: AACTCACCAGATGCCAAGTCGAAG  R: TGCTAGAAGCAACTTTCTCACCACG |
| Phvul.004G015800 | F: GACAGGCTCGCTTCTCCTCAATT  R: GTTGGGATCCAAGTTGTCTCTCAAG |
| Phvul.008G164300 | F: CCTTACAAACTGCCAGCAGAAG  R: CAATATCTTCCGAAGACCCATCCC |
| Phvul.005G162600 | F: GAGCAACCATCATGAGCACCG  R: CCACCCTGATTCACCGTTCCTC |
